# Detection of a Hb A_2_‐Melbourne (HBD: c.130G>A) combined with β‐thalassemia in a Chinese individual

**DOI:** 10.1002/jcla.23401

**Published:** 2020-08-08

**Authors:** Youqiong Li, Tongfeng Huang, Tian Mao, Xiuqun Zhang, Liang Liang, Menghui Meng

**Affiliations:** ^1^ Center of Medical Genetics and Prenatal Diagnosis People’s Hospital of Guangxi Zhuang Autonomous Region Nanning China; ^2^ Department of Laboratory Medicine Chongzuo Maternal and Child Health Hospital Chongzuo China

**Keywords:** capillary electrophoresis (CE), Hb A_2_‐Melbourne, high‐performance liquid chromatography (HPLC), thalassemia, δ‐globin gene mutation

## Abstract

**Background:**

Thalassemia is common in Southeast Asian countries, including China. Hb A_2_‐Melbourne is a rare hemoglobin variant and has never been reported in China. Here, we report a Hb A_2_‐Melbourne combined with β‐thalassemia in Chinese individuals which is the second case described in the published reports.

**Methods:**

Complete blood counts (CBC) of a 28‐year‐old female showed signs of thalassemia during a routine screening. Hemoglobin analysis was subsequently performed using capillary electrophoresis (CE) and high‐performance liquid chromatography (HPLC). Four common deletional α‐thalassemia detection was carried out using a gap‐polymerase chain reaction (PCR). PCR and reverse dot‐blot were used to detect three non‐deletional α‐thalassemia and 17 types of point mutations in β‐thalassemia. Finally, it was identified by Sanger sequencing. Her husband also had CBC, hemoglobin analysis, and genetic diagnosis.

**Results:**

CBC of the couple showed Hb 103 and 139 g/L, mean corpuscular volume 58 and 63.1 fL, mean corpuscular hemoglobin 19.7 and 20.4 pg, respectively. Hemoglobin analysis revealed Hb X 2.4%, Hb A_2_ 2.8% by CE and Hb X 2.9%, Hb A_2_ 2.4% by HPLC in the female. The results of her husband were Hb A93.5%, Hb A2 5.7%, Hb F 0.8% by CE. Genetic analysis of both spouses detected the same CD 41/42 mutations in β‐globin gene. Sanger sequencing of female identified a mutation of the δ‐globin gene (*HBD:*c.130G>A), corresponding to Hb A_2_‐Melbourne.

**Conclusion:**

Hb A_2_‐Melbourne can lead to misdiagnosis of β‐thalassemia. δ‐globin gene mutation must be carefully examined in routine thalassemia screening.

## 1 INTRODUCTION

β‐thalassemia is one of the most common hereditary disorders in the world.^1^ In southern China, the carrier rate of β‐thalassemia is high with a prevalence of 6.43% in Guangxi and 2.54% in Guangdong.^2^, ^3^ Hb A_2_, composed of two α chains and two δ chains, is a minor component of the hemoglobin present in normal adult red blood cells, accounting for 2.5%‐3.5% of the total hemoglobin in healthy individuals.^4^ Increased Hb A_2_ is the most significant characteristic in β‐thalassemia carriers.^4^ Therefore, Hb A_2_ determination plays a key role in screening for β‐thalassemia. However, some individuals with silent β‐globin gene mutations or with triplicate α‐globin genotypes (ααα/αα) may be misdiagnosed during screening programs which show normal or reduced Hb A_2_.^5^ Besides, δ‐globin gene mutation may influence the Hb A_2_ levels in the blood. Individuals with compound heterozygotes β‐thalassemia with a δ‐globin gene mutation may have normal Hb A_2_ levels and therefore be overlooked as β‐thalassemia heterozygotes. Twenty‐one δ‐globin gene mutations were detected in the Chinese population. However, Hb A_2_‐Melbourne has not yet been described.^6^ When variants of the δ‐globin gene are not accurately identified and not counted into the total Hb A_2_, then β‐thalassemia also may be misdiagnosed. Herein, this study is the first time that Hb A_2_‐Melbourne combined with β‐thalassemia has been reported in Chinese population.

## 2 METHODS

### 2.1 Complete blood counts and hemoglobin analysis

This study was approved by the Ethics Committee of Guangxi Zhuang Autonomous Region People's Hospital. Signed informed consent was obtained from patients.

A 28‐year‐old female and her husband visited our hospital for a routine prenatal examination. According to our hospital process, they were asked to conduct thalassemia screening by complete blood counts (CBC) and hemoglobin analysis. CBC were analyzed on an automated cell counter (Sysmex Xi‐2000, Sysmex Co.). Hemoglobin analysis was performed using capillary electrophoresis (CE) method (Capillarys 2, Sebia) according to the manufacturer's instructions. At the same time, high‐performance liquid chromatography (HPLC) (VARIANT II™, Bio‐Rad) was also performed as comparison experiment.

### 2.2 Routine thalassemia genetic analysis

The four common α‐thalassemia deletions Southeast Asian (‐‐^SEA^/), Thailand (‐‐^THAI^/), 3.7 kb (rightward) (‐α^3.7^/) and 4.2 kb (leftward) (‐α^4.2^/) in the Chinese population were carried out using routine gap‐polymerase chain reaction (gap‐PCR) (Shenzhen Ying Sheng‐tang Bio‐Tech Ltd.). PCR and reverse dot‐blot and PCR (RDB‐PCR) were performed for three common α‐thalassemia point mutations: Hb Constant Spring (Hb CS), Hb Quong Sze (Hb QS), and Hb Westmead (Hb WS) (Shenzhen Yaneng Bio‐Tech Ltd.). The common β‐thalassemia mutations in Chinese population (−28M, −29M, −30M, −32M, CDs 14/15M, CD17M, CD 26M, CDs 27/28M, CD 31M, CDs 41/42M, CD 43M, CDs 71/72M, IVS‐I‐1M, IVS‐Ⅰ‐5M, IVS‐II‐654M, CAP +1M, initiation codon M) were also performed by RDB‐PCR (Shenzhen Yaneng Bio‐Tech Ltd.).

### 2.3 Sanger DNA sequencing

As previously described by Dongzhi Li et al, two pairs of primers were designed to sequence the δ‐globin gene.^6^ The first amplified fragment (897 bp) was from position −219 of the cap site to +678 (Forward primers 5′‐AGA TGC GGT GGG GAG ATA‐3′ and reverse primers 5′‐TAG CAA GAT TGT GAG GAA GGA A‐3′). The second fragment (470 bp) was from position +1310 to +1779 (Forward primers 5′‐GGG TGT TGG CTC AGT TTC‐3′ and reverse primers 5′‐GTA CGG TTC CCT TGC TTT‐3′). DNA was amplified using Taq polymerase (Promega) in C‐1000 Thermal Cycler (Bio‐Rad). PCR was performed in 50 µL‐reaction including 0.5 U La Taq polymerase with an annealing temperature of 62°C for 1 minutes and 30 cycles. The PCR products were sequenced by the 3730 automated sequencer (Applied Biosystems).

## 3 RESULTS

### 3.1 Hematological analysis

**Figure 1:**
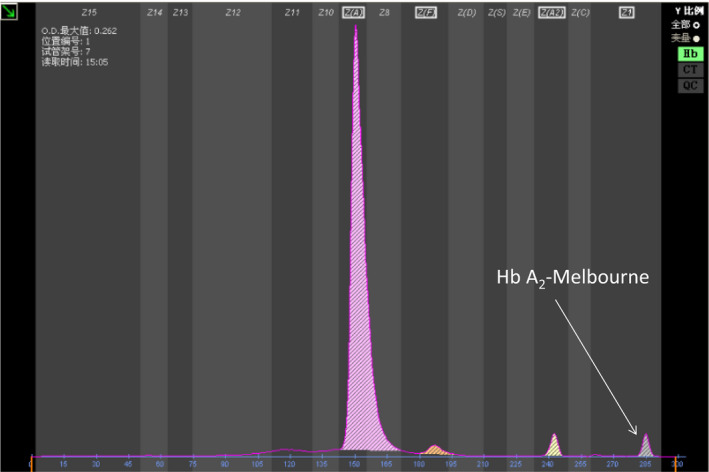
Hb analysis of the female using CE. Hb A_2_‐Melbourne was indicated in Zone 1

**Figure 2:**
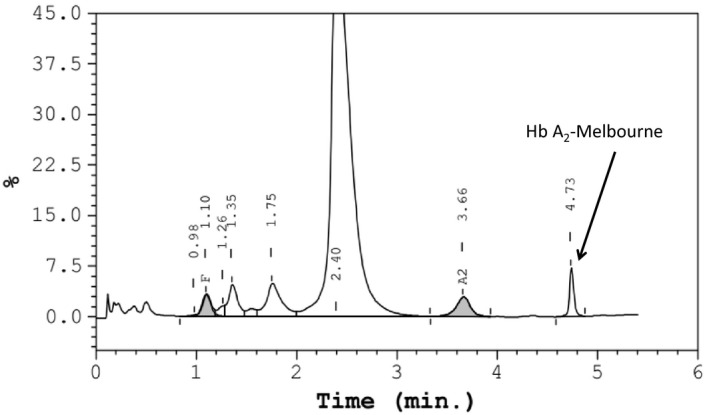
Hb analysis of the female using HPLC. An abnormal peak (Hb A_2_‐Melbourne) was observed at an RT of 4.73 min

Red cell indices of the female were the following: Hb 103 g/L (ref: 110‐150 g/L), mean corpuscular volume (MCV) 58.0 fL (ref: 80.0‐99.0 fL), mean corpuscular hemoglobin (MCH) 19.7 pg (ref: 27‐32 pg). Hemoglobin analysis using CE showed the quantity of different fractions: Hb A 93.4% (ref: 96.5%‐97.5%), Hb A_2_ 2.8% (2.4%‐3.4%), Hb F 1.5% (0%‐5%), Hb A_2_ variant 2.4% (zone 1) (Figure 1). Results of HPLC were Hb F 2.2%, Hb A_2_ 2.9%, and an abnormal peak (2.4%) with an RT of 4.73 minutes (Figure 2). Her husband had typical hematological results of β‐thalassemia trait: HGB 139 g/L, MCV 63.1 fL, MCH 20.4 pg, Hb A 93.5%, Hb A_2_ 5.7%, Hb F 0.8% (Table 1).

**Table 1:** Hematological parameters, hemoglobin analysis, and globin genotypes of the couples

Parameters	Female	Her husband
Age (years)	28	29
Hb (g/dL)	103	139
MCV (fL)	58.0	63.1
MCH (pg)	19.7	20.4
Hb A (%)	93.4	93.5
Hb F (%)	1.5	0.8
Hb A2 (%)	2.8	5.7
Hb A_2_‐Melbourne	2.4	0
α Genotype	αα/αα	αα/αα
β Genotype	β^CD41/42^/β^N^	β^CD41/42^/β^N^
δ Genotype	δ^CD43^/δ^N^	δ^N^/δ^N^

### 3.2 Routine thalassemia genetic analysis and DNA sequencing

**Figure 3:**
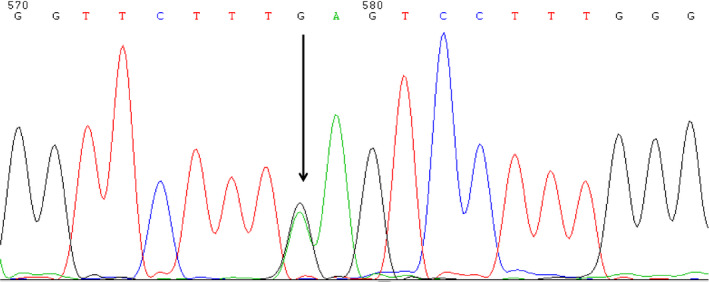
DNA sequencing revealed a heterozygous for GAG > AAG mutation at codon 43 of the δ‐globin gene, corresponding to a Hb A_2_‐Melbourne

RDB‐PCR of female showed a CD 41/42 mutations in β‐globin gene but none among the three common α‐thalassemia mutation. Her husband had the same mutation in β‐thalassemia. DNA sequencing of female revealed the GAG > AAG mutation at codon 43 of δ‐globin gene corresponded to the Hb A_2_‐Melbourne [δ43(CD2) Glu → Lys (GAG > AAG)] (Figure 3).

## 4 DISCUSSION

Mutations that occur on the δ‐globin gene can affect the structure or the expression of the δ‐globin chain. It will produce a second and usually visible Hb A_2_ variant with stable structural defects (as Hb A_2_‐Melbourne in this study). If the structure of the variant is unstable, the mutation will behave as a thalassemic defect (δ‐thalassemia) and be undetectable using CE or HPLC.^7^ Although most of the δ‐globin variants are pathologically innocuous, coinheritance with β‐thalassemia or α‐thalassemia could lead to misdiagnosis.^8^, ^9^ The coexistence of a δ‐globin gene defect will decrease the Hb A_2_ level of the β‐thalassemia or α‐thalassemia carrier to a normal range.^10^ Therefore, it is essential to be aware of the existence of δ‐globin gene defect in routine thalassemia screening.

As shown in the Figures 1, 2, in addition to Hb A_2_, a small peak of an abnormal Hb variant was observed on both CE and HPLC. Based on its value and location, we speculated that it is an Hb A_2_ variant. So, the value of Hb A_2_ variant should be added to the total Hb A_2_ value (Hb A_2_ of CE: 2.8% + 2.4% = 5.2%, Hb A_2_ of HPLC: 2.9% + 2.4% = 5.3%) which was an increased Hb A_2_ level. With her red cell indices, it suggested that female was a suspected Hb A_2_ variant coinheritance with β‐thalassemia. Finally, routine thalassemia genetic analysis and DNA sequencing were verified that the female had both Hb A_2_‐Melbourne and a CD 41/42 mutations in β‐globin gene. Hb A_2_‐Melbourne was originally described in an Italian family and reported in Thailand and Laos population, but not previously described in China. ^11^, ^12^, ^13^ Unfortunately, her husband was a β‐thalassemia trait (CD 41/42 mutations in β‐globin gene) by routine thalassemia genetic analysis. It means that their next generation will have risk having homozygotes β‐thalassemia. Because of the accurate identification in our laboratory, the couple obtained the genetic counseling timely.

Small peaks appear in the electrophoresis, which may be unstable hemoglobin, degraded hemoglobin, or Hb A_2_ variant. The recognition of Hb A_2_ variant is important for the diagnosis and exclusion of β‐thalassemia or α‐thalassemia. Therefore, when additional small peaks occur near the Hb A_2,_ one should first determine whether it is an Hb A_2_ variant. In most cases, experienced technicians can determine by the characteristics of electrophoresis. As shown in CE and HPLC, it generally migrates behind the Hb A_2_ and value is usually below that of the Hb A_2_. Our findings are similar to previous study but it has equal proportions of Hb A_2_ and Hb A_2_‐Melbourne in double heterozygote for Hb A_2_‐Melbourne/α^+^‐thalassemia.^10^ Once the Hb A_2_ variant is determined, Hb A_2_ and Hb A_2_ variant must be summed up to calculate the real Hb A_2_ level ( total Hb A_2_ = Hb A_2_ + Hb A_2_ variant). Otherwise, as in this study, coinheritance of Hb A_2_‐Melbourne may lead to a missed diagnosis of β‐thalassemia trait by causing normal Hb A_2_ level if value of Hb A_2_‐Melbourne was not counted into the total Hb A_2_.

## CONFLICT OF INTEREST

No potential conflict of interest was reported by the authors.
